# Spatial, temporal, and spatiotemporal analysis of mumps in Guangxi Province, China, 2005–2016

**DOI:** 10.1186/s12879-018-3240-4

**Published:** 2018-08-02

**Authors:** Guoqi Yu, Rencong Yang, Yi Wei, Dongmei Yu, Wenwen Zhai, Jiansheng Cai, Bingshuang Long, Shiyi Chen, Jiexia Tang, Ge Zhong, Jian Qin

**Affiliations:** 1 Department of Environmental and Occupational Health, Guangxi Medical University, Nanning, Guangxi Zhuang Autonomous Region China; 2 Guangxi Center for Disease Control and Prevention, Institute of Vaccination, Nanning, Guangxi Zhuang Autonomous Region China; 3 Department of Health Related Social and Behavioral Science, West China School of Public Health, Sichuan University, Chengdu, Sichuan China

**Keywords:** Mumps, Guangxi, Spatial analysis, Cluster

## Abstract

**Background:**

The resurgence of mumps around the world occurs frequently in recent years. As the country with the largest number of cases in the world, the status of mumps epidemics in China is not yet clear. This study, taking the relatively serious epidemic province of Guangxi as the example, aimed to examine the spatiotemporal pattern and epidemiological characteristics of mumps, and provide a scientific basis for the effective control of this disease and formulation of related health policies.

**Methods:**

Geographic information system (GIS)-based spatiotemporal analyses, including spatial autocorrelation analysis, Kulldorff’s purely spatial and space-time scan statistics, were applied to detect the location and extent of mumps high-risk areas. Spatial empirical Bayesian (SEB) was performed to smoothen the rate for eliminating the instability of small-area data.

**Results:**

A total of 208,470 cases were reported during 2005 and 2016 in Guangxi. Despite the fluctuations in 2006 and 2011, the overall mumps epidemic continued to decline. Bimodal seasonal distribution (mainly from April to July) were found and students aged 5–9 years were high-incidence groups. Though results of the global spatial autocorrelation based on the annual incidence largely varied, the spatial distribution of the average annual incidence of mumps was nonrandom with the significant Moran’s *I*. Spatial cluster analysis detected high-value clusters, mainly located in the western, northern and central parts of Guangxi. Spatiotemporal scan statistics identified almost the same high-risk areas, and the aggregation time was mainly concentrated in 2009–2012.

**Conclusion:**

The incidence of mumps in Guangxi exhibited spatial heterogeneity in 2005–2016. Several spatial and spatiotemporal clusters were identified in this study, which might assist the local government to develop targeted health strategies, allocate health resources reasonably and increase the efficiency of disease prevention.

**Electronic supplementary material:**

The online version of this article (10.1186/s12879-018-3240-4) contains supplementary material, which is available to authorized users.

## Background

Mumps is an acute communicable disease caused by the mumps virus [1]. As a highly contagious disease, mumps is characterized by the clinical manifestations that swelling of the parotid glands, accompanied with pain and fever. Although this infection in the majority cases is mild, its complications, such as meningitis. Orchitis, ovarian inflammation, and deafness, affecting the nervous system, digestive system and causing other multiple organ damages are serious [2]. In most parts of the world, the annual incidence of mumps in the absence of immunization ranges from 100 to 1000 cases/100,000 population, with epidemic peaks every 2–5 years [3]. The incidence of mumps is rapidly declining because of the widespread use of vaccine, especially in combination with vaccines for measles and rubella (measles–mumps–rubella [MMR] vaccine). The United States, Australia, and many European countries such as the United Kingdom, Italy, etc. have conducted routine administration of 2 doses of MMR vaccine for children since the last century [4–7]. Nevertheless, mumps resurgences have been observed in many countries, even in those countries with higher immunization rates since the beginning of the new century [8–11]. This phenomenon may be due to variation in the virus strain, periodicity of the disease, attenuation of the body antibody and other factors [12–14]. Studies found that using three doses of MMR vaccination can significantly increase the crowd’s immunity to mumps [7, 15]. In addition, increasing the coverage of vaccination and timely supplementary immunization activities (SIA) in high-risk areas are also keys to preventing mumps outbreak. China, the world’s most populous country, has the largest number of reported mumps cases, which accounted for approximately 52.19% in Asia-Pacific countries and 30.01% in the world in 2016 [16]. Monovalent vaccine for mumps has been applied in China since the 1990s. In 2008, a vaccine containing the component of mumps was included in the expanded program on immunization (EPI). Children between the ages of 18 and 24 months can receive a free dose of MMR. In this way, the mumps epidemic has been greatly weakened due to the reduction of susceptible population. However, only a few regions have implemented two doses MMR immunization plan in China. The incidence of mumps in some provinces, such as Guangxi, Ningxia, and Hainan, is higher than the national average because of the low immunization rate. Previous studies on mumps in China had focused on its epidemiological characteristics rather than its spatial and temporal distribution. Spatiotemporal analysis on mumps in local areas mainly concentrated in the northern parts of China, such as Shandong, Gansu, and Shanxi [17, 18]. The economy, climate, and geography of Guangxi, as a high-incidence area of mumps and mountainous province in southern China, differ from those of the northern region of China. Therefore, Guangxi was chosen as the ideal site for spatiotemporal analysis in this study.

Many infectious diseases, including malaria, measles, Japanese encephalitis, and mumps, exhibit heterogeneity in spatial and temporal distribution [19–22]. Spatial statistical methods, based on the geographical information system (GIS), have been widely applied for their strong statistical power in the surveillance of communicable diseases and the evaluation of the effectiveness of preventive interventions in the past decades [23, 24]. Spatiotemporal analysis can not only visualize the epidemiological data and help observe the geographical distribution of disease from a more intuitive point of view, but identified spatial clusters and temporal clusters from a deeper level and determine the high- and low-risk areas. People inhabiting high-risk areas are the target population for health agencies to implement SIAs.

In this study, spatial autocorrelation and spatiotemporal scan analyses were conducted to statistically evaluate the significance of aggregation and determine the size of the range of hot-spots at the county level in 2005 and 2016. The results of these analysis could enhance the understanding of the epidemic characteristics and spatiotemporal patterns of mumps in Guangxi and provide a scientific basis for policy formulation and health resource allocation.

## Methods

### Study area

**Fig. 1:**
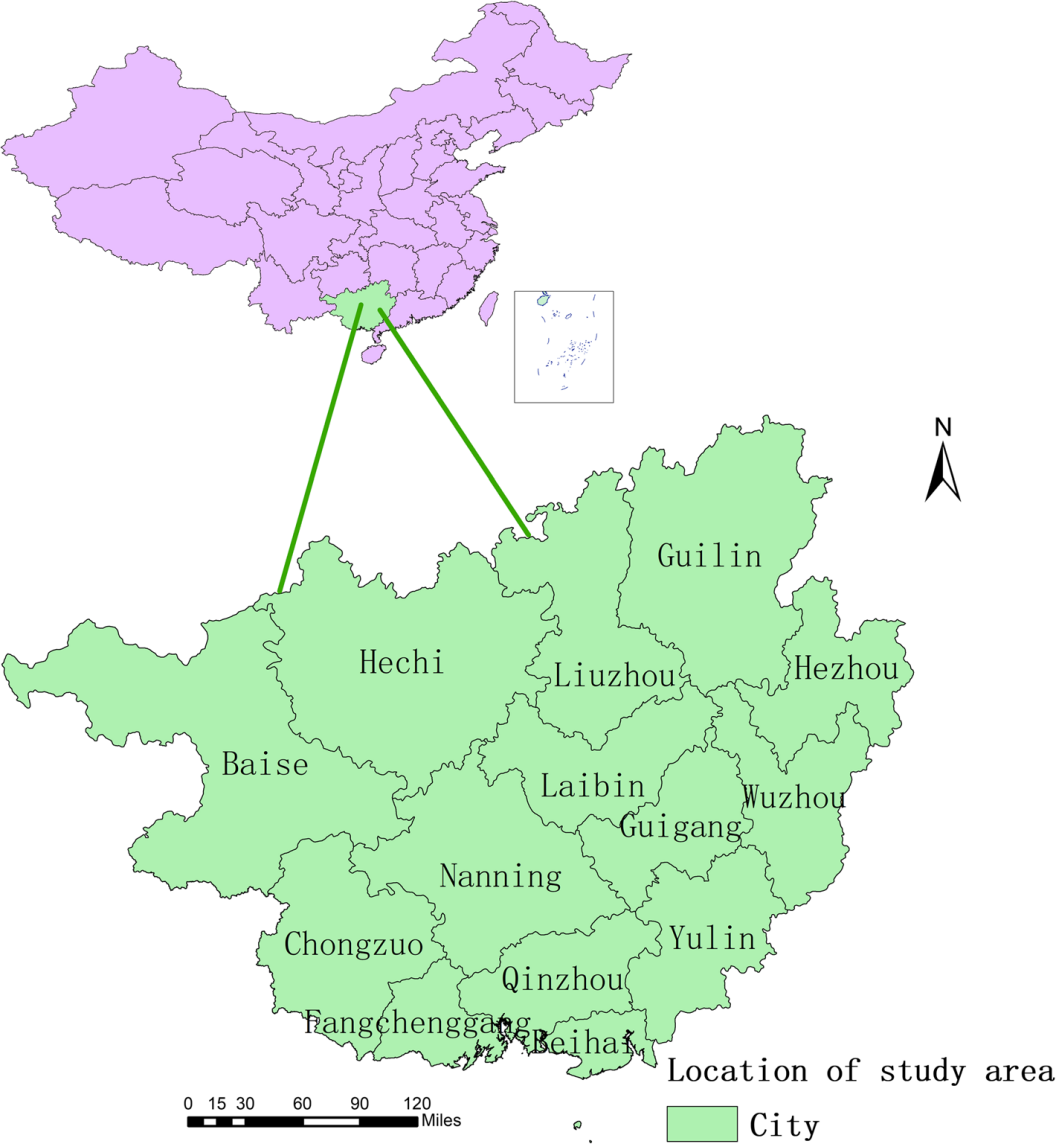
The geo-location of Guangxi Province in China. The map was created with ArcGIS software version 10.4 (ESRI, Inc., Redlands, CA, USA)

The study site is the Guangxi Zhuang Autonomous Region (104° 26′–112° 04′ E, 20° 54′–26° 24′ N), which is located in the southwestern part of China, adjacent to the South China Sea. It is a mountainous province with a subtropical monsoon climate. Abundant rainfall, warm climate and not obvious seasonal changes are its main climate characteristics. Guangxi, with 14 administrative cities and 109 counties or urban districts, has a land area of 236,700 km^2^ and a population of 47.96 million by the end of 2016. All of these areas were included in this study (Fig. 1).

### Data collection and management

Data on the monthly mumps cases between 2005 and 2016 were obtained from the China Information System for Disease Control and Prevention (CISDCP). Information on the notified cases included case number; county code; date of symptom onset; and demographic characteristics, such as gender, age, occupation, and residential address. All mumps cases were identified and diagnosed according to the criteria established in the “Diagnostic criteria for mumps” published by the Chinese Ministry of Health. The diagnosis of the case was based on clinical manifestations and associated laboratory test results, and the diagnostic criteria for the disease were consistent during the study period. Confirmed or suspected cases were reported within 1 day through the network or an infectious disease report card. The reported data were checked and evaluated by qualified personnel. Population data were derived from the Information Management Section of the Guangxi Center for Disease Control and Prevention. A vector map of Guangxi at the county level was collected from the Guangxi Bureau of Surveying, Mapping and Geoinformation. All mumps cases were geocoded and matched to the county level polygons by the specific administrative code.

In order to make sure that the reported cases were newly emerged, we re-checked the obtained data and deleted the repeated cases that were detected in our study. Cases with unknown county (district) of residence were also excluded. According to the registration information of infectious diseases, we describe the distribution characteristics of mumps cases such as sex, age, occupation, seasonality and whether the mumps cases are imported cases. Among them, the age of the mumps cases was divided into nine groups. Several statistical indicators in the research can be calculated as follows:

- The annual morbidity: the annual number of cases/the annual population*100,000;
- The average sex ratio: the total male cases/the total female cases;
- The average annual number of cases: the total number of cases/12;
- The average annual incidence: the average annual number of cases/the average population*100,000;

### Spatial smoothing using empirical Bayesian analysis

The data for the spatial-temporal analysis of the study based on a county level are small area data, barely relying on them could make the results shaky. In order to reduce the random error caused by small area data and rare events, Spatial Empirical Bayesian (SEB) was used to make the results more reliable and stable. This process of smoothing the average annual incidence and the annual incidence of each county between 2005 and 2016 was conducted by specifying a spatial weights file generated by the algorithm of k-nearest neighbors. The Bayesian model will allow the morbidity of counties with small population at risk to be appropriately adjusted, whereas it has less impact on the raw rates of big areas [25].

### Spatial autocorrelation analysis

Describing the spatial characteristics of the disease is one of the prerequisites for risk factor analysis. Spatial autocorrelation analysis is an important process involved in this descriptive analysis. This process mainly includes global autocorrelation and local autocorrelation. The former can be used to analyze whether the attributes specified in an entire study area are relevant at the county level, while the later can accurately determine where such attributes are gathered and reveal the spatial distribution pattern and the approximate spatial aggregation range. Moran’s index (Moran’s *Ι*), which generally ranges from − 1 to + 1, is an important indicator of global autocorrelation analysis. A value close to 1 or to − 1 indicates a strong positive or negative spatial autocorrelation. Moran’s *I* can be tested based on their Z-scores and *P*-value, which can determine whether or not to reject the null hypothesis that the incidence of mumps was randomly distributed in space [26]. In the hypothesis test of the local indicators of spatial autocorrelation (LISA), local autocorrelation can exist only when |z| ≥ 1.96 and *P* ≤ 0.05 (α = 0.05). The association pattern can be divided into four categories: High-High; High-Low; Low-Low; Low-High. All the autocorrelation analysis was based on the smoothing rate calculated by specifying a weight file.

### Spatial and space-time cluster analysis

Spatial autocorrelation analysis can determine the spatial heterogeneity of a disease, whereas has limited function in detecting the specific aggregation range of the disease and its clustering in time. Thus, two spatial scan statistics were performed in this study: purely spatial cluster analysis and spatiotemporal cluster analysis. Both of them were fitted by a discrete Poisson model. The former imposed a circular window on the map, which in turn was centered on each of the several possible grid points positioned throughout the study region. The latter was defined by a cylindrical window with a circular (or elliptical) geographic base and with a height corresponding to time. The base was defined exactly as that of purely spatial scan statistics, whereas the height reflected the period of potential clusters. The cylindrical window was then moved in space and time, so that for each possible geographical location and size, it also visited each possible time period [27]. The maximum spatial cluster size was set to 10% of the population at risk in the spatial window, and a maximum temporal cluster size of 20% of the study period and a minimum temporal cluster size of 1 month were assigned in the temporal window in the study, respectively. No geographical overlap was allowed in reporting secondary clusters. The log likelihood ratio (LLR) was mainly used to determine the most likely cluster and the secondary clusters. The relative risk (RR), which is the estimated risk within the cluster divided by the estimated risk outside the cluster, is an important indicator to assess the risk of each cluster. In this study, the number of Monte Carlo simulation was set to 999 and the statistically significant level was set as 0.05 [28].

SEB and LISA were performed in GeoDa software (version 1.12). The purely spatial scan and the space-time scan proposed by Kulldorff were completed in SaTScanTM version 9.4. All the results of spatial analysis were visualized by using ArcGIS software version 10.4 (ESRI, Inc., Redlands, CA, USA).

## Results

### Epidemiological features

#### General demographic characteristics

**Fig. 2:**
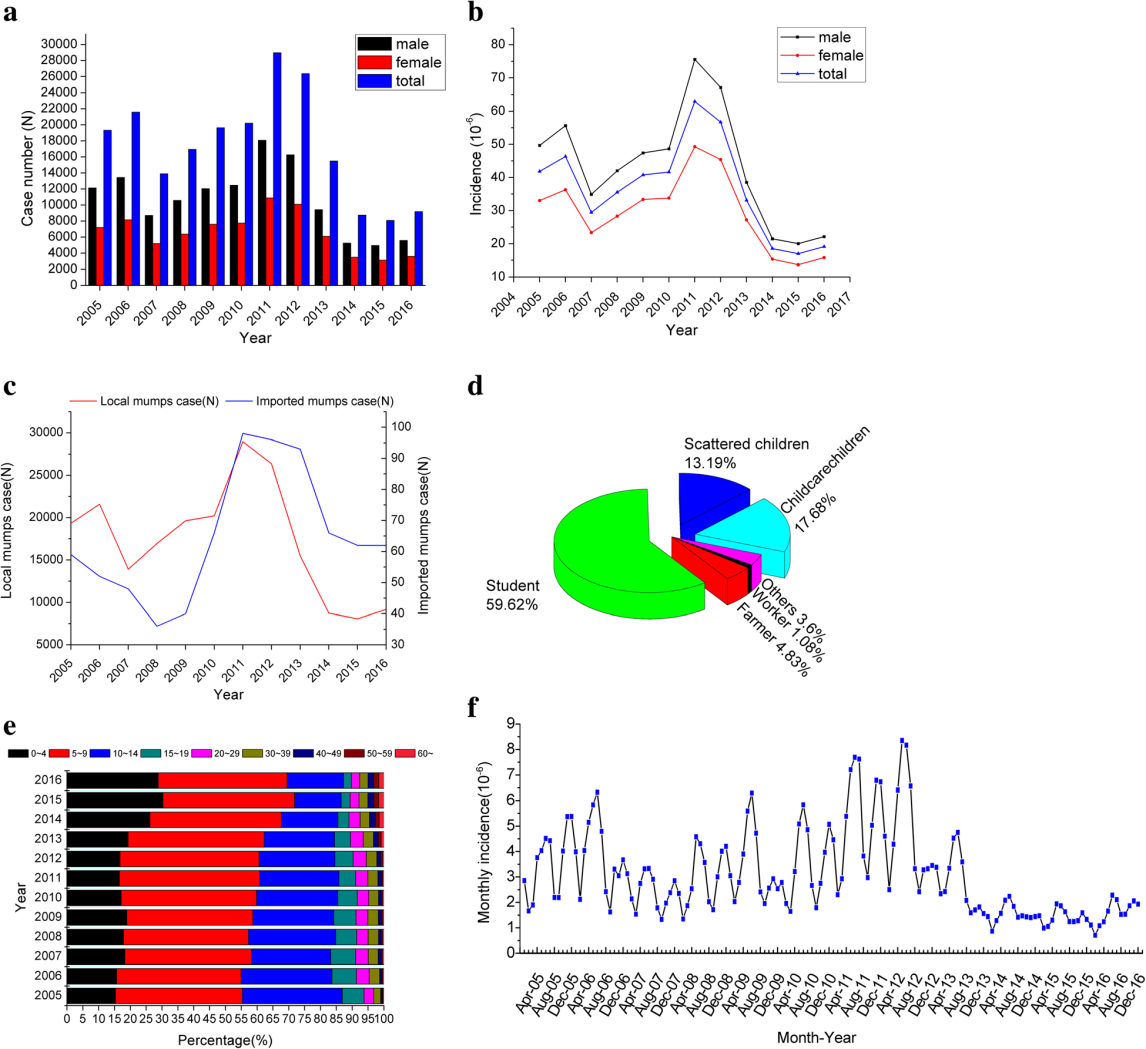
Epidemiological characteristics of mumps in Guangxi from 2005 to 2016. **a** Annual case number of mumps in different gender; **b** Annual incidence of mumps in different gender; **c** Case source distribution of mumps; **d** School age and occupation distribution of mumps; **e** Age distribution of mumps; **f** Monthly distribution of mumps

A total of 208,470 mumps cases were included in this study between 2005 and 2016. In general, the annual case number and the annual morbidity of mumps maintain a similar trend in the study period, with ranging from 8078 to 28,983 and from 16.99 (per 100,000) to 62.97 (per 100,000), respectively. Two peaks were observed in 2006 and 2011, and the incidence rate increased again after 2015. The incidence of males was always higher than that of females and was consistent with the trend of overall morbidity (Fig. 2a and b). The average sex ratio of the incidence was 1.47:1. The local cases of mumps accounted for a majority (99.63%) compared to the imported cases (0.27%) and the fluctuation of the cases between them followed a similar pattern (Fig. 2c). Students were the main group at risk of mumps, followed by childcare children and scattered children (Fig. 2d). Children aged 5–9 years old obtained the highest reported incidence, which ranged from 92.34(per 100,000) to 408.82(per 100,000) in the study period, followed by the age group of 10–14 years old [41.05(per 100,000)–219.81(per 100,000)]. The proportion of the case number and the incidence in the older age group (i.e., 50–59 years old and 60 and above) showed an overall increasing trend, whereas the incidence of other age groups exhibited a decreasing trend after 2011 (Fig. 2e, Additional file 1: Figure S1).

#### Seasonal pattern

The distribution of mumps displayed a clear seasonal pattern. Two significant peaks were observed in the study period: the first peak was from April to July, whereas the second peak was from October to January in the next year. The highest number of cases always concentrated in May or June each year. After 2013, the seasonal trend of mumps was not as apparent as before (Fig. 2f).

#### Geographical distribution

**Fig. 3:**
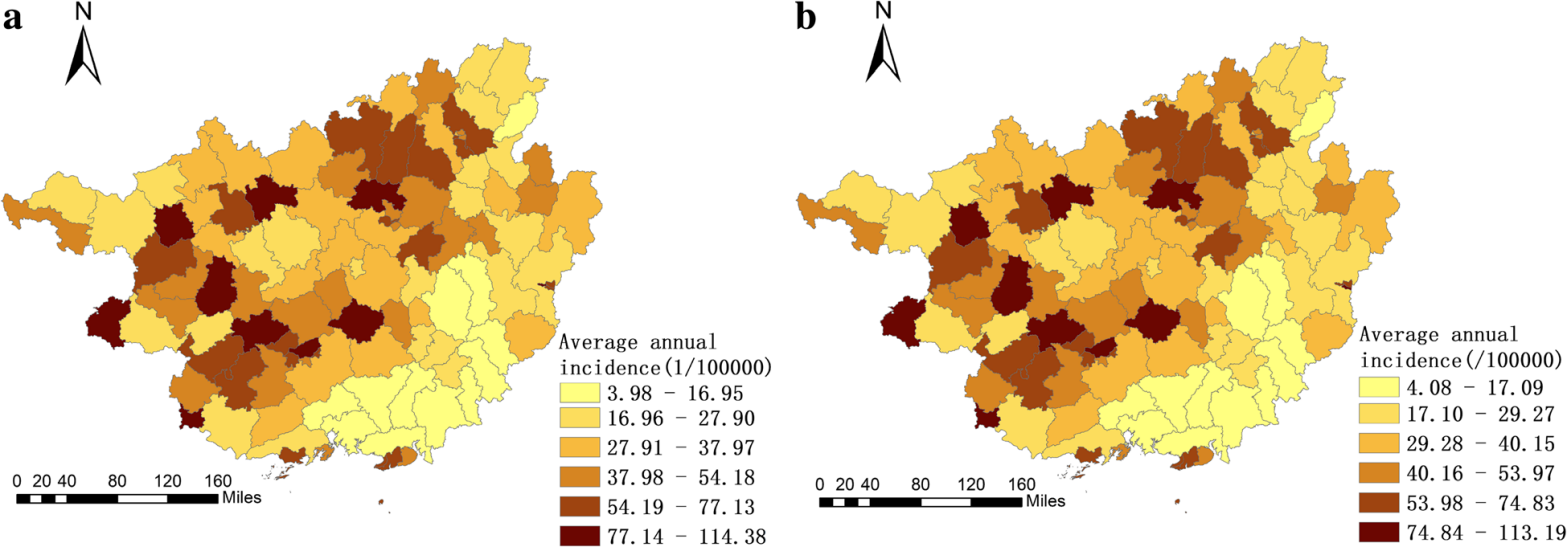
Average annual incidence at county level in Guangxi from 2005 to 2016. **a** The raw rate of average annual incidence; **b** The average annual incidence smoothed by the Spatial Empirical Bayesian (SEB)

**Fig. 4:**
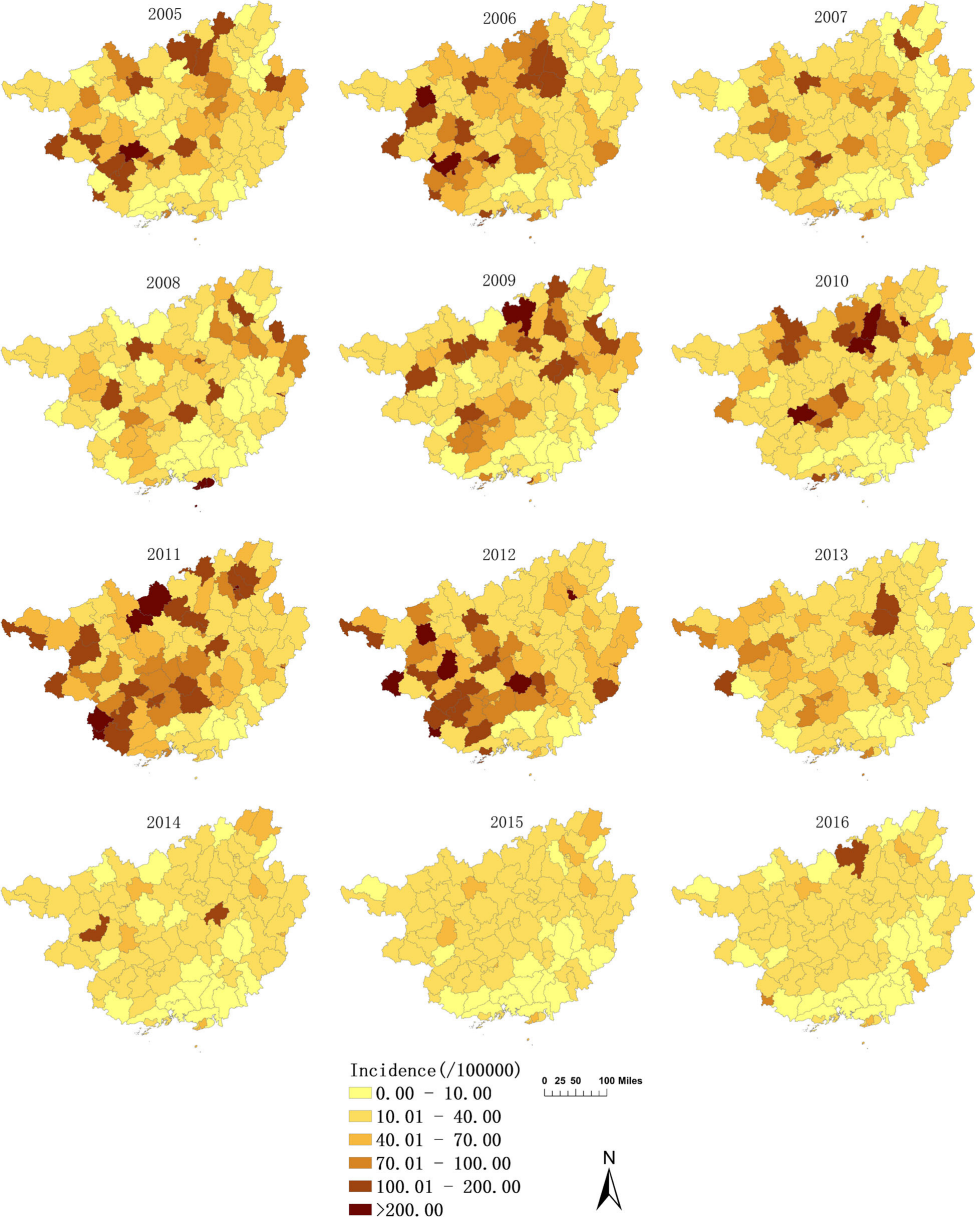
Annual incidence rates of mumps in Guangxi, China at county level from 2005 to 2016

As shown in Fig. 3, the high incidence of mumps mainly concentrated in the western and northern parts of Guangxi. The average annual incidences of Lingyun, Jinchengjiang, Napo, Tiandong, Longan, Pingxiang, Xingning, Liucheng and Binyang were higher than those of other counties (districts). In addition, significant changes in the average annual incidence after SEB smoothing were not observed compared with the raw rates. Both of them showed almost the same distribution pattern geographically. The thematic map of the annual incidence indicated that the northern and western parts of Guangxi were still the high incidence regions of mumps, especially some counties in the cities of Nanning, Liuzhou, Chongzuo and Baise. Furthermore, 2005, 2006, 2009, 2010, 2011, and 2012 were the relatively severe years of mumps epidemic. Nevertheless, the overall epidemic occurrence has declined since 2012. The annual incidence of mumps decreased rapidly, and the regional differences became less evident (Fig. 4).

### Cluster analysis

The global spatial autocorrelation analysis of the average annual incidence of mumps in Guangxi suggested that a significant positive spatial autocorrelation existed. Similar statistically significant results were also observed when the analysis was conducted year by year, except in 2007, 2008, 2014, and 2016 (Table 1).

**Table 1:** The global spatial autocorrelation of mumps in Guangxi, China, 2005–2016

Year	Moran *I*	*Z*-value	*P*-value	E[I]	Mean	SD
2005	0.132	3.43	0.003	−0.009	−0.009	0.041
2006	0.136	3.37	0.003	−0.009	−0.005	0.042
2007	0.021	0.74	0.230	−0.009	−0.010	0.042
2008	−0.016	−0.22	0.461	−0.009	− 0.007	0.042
2009	0.070	1.88	0.038	−0.009	−0.010	0.043
2010	0.180	4.74	0.001	−0.009	−0.010	0.040
2011	0.126	3.30	0.004	−0.009	−0.011	0.042
2012	0.081	2.22	0.003	−0.009	−0.009	0.040
2013	0.086	2.30	0.021	−0.009	−0.010	0.042
2014	0.056	1.65	0.063	−0.009	−0.010	0.040
2015	0.088	2.31	0.020	−0.009	−0.009	0.042
2016	−0.032	−0.57	0.304	−0.009	− 0.009	0.041
Average	0.128	3.18	0.004	−0.009	−0.010	0.043

Note: E[I]: the theoretical mean of Moran *I* statistic. Mean and SD: the centralized and discrete trends of simulated empirical distribution

**Fig. 5:**
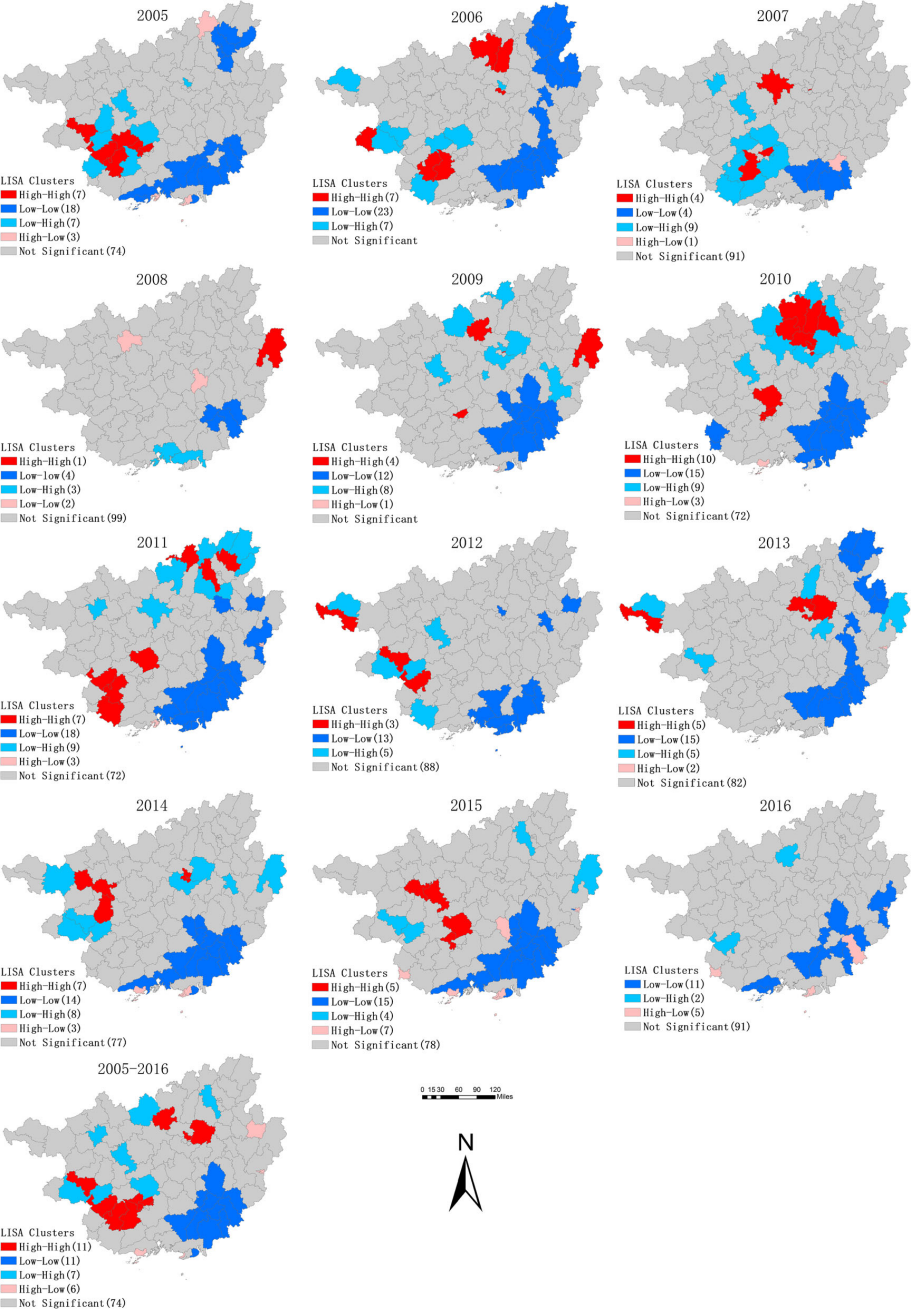
Yearly Local Indicators of Spatial Association (LISA) cluster maps for mumps incidence, Guangxi, 2005–2016

LISA analysis can effectively identify the hot spots (High-High) and cold spots (Low-Low) of mumps, although the locations of the hot spots were in a continuous transfer, they were mainly in the northern and western parts of Guangxi. Xingning, Jiangnan, Wuming, Yufeng, Liunan, Jiangzhou and Jiangnan owned more times of hot spots between 2005 and 2016. Almost all of these hot spots belong to the urban areas of each city. Two thousand ten was the year with the most hotspots (Table 2). Cold spots stably existed in the southeastern part of Guangxi. In addition, 11 hot spots and 11 cold spots were identified in the LISA analysis of the average annual incidence at the county level. Hot spots mainly concentrated in Liuzhou, Chongzuo and Nanning, which were also the regions that need us attach more attention to (Fig. 5).

**Table 2:** Hot spots summary resulting from the local indicators of spatial analysis (LISA) between 2005 and 2016

Year	Hot Spots of High-High aggregation
2005	Debao, Longan, Daxin, Xixiangtang, Xingning, Jaingnan, Jiangzhou
2006	Rongshuimiao, Rongan, Liunan, Yufeng, Napo, Jiangzhou, Fusui
2007	Yizhou, Fusui, Xingning
2008	Babu
2009	Luocheng, Babu, Xingning
2010	Wuming, Xingning, Jiangnan, Rongshuimiao, Luocheng, Rongan, Liucheng, Yongfu, Liubei, Yufeng
2011	Wuming, Daxin, Jiangzhou, Ningming, Sanjiang, Lingui, Xingan
2012	Xilin, Debao, Daxin
2013	Xilin, Liucheng, Luzhai, Liunan, Yufeng
2014	Lingyun, Tiandong, Bama, Liubei, Liunan, Yufeng
2015	Bama, Dahua, Wuming, Xixiangtang, Jiangnan
2016	none
Average	Luocheng, Luzhai, Liubei, Liuzhong, Debao, Daxin, Jiangzhou, Fusui, Xixiangtang, Xingning, Jiangnan

**Fig. 6:**
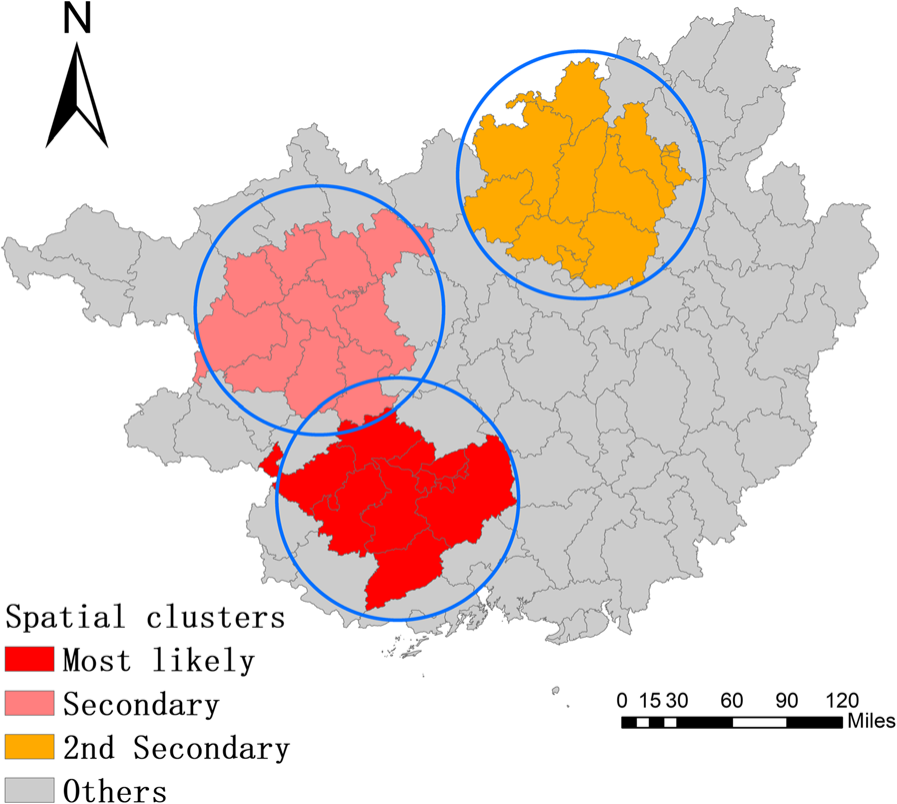
Spatial clusters of mumps at county level in Guangxi, China from 2005 to 2016

Kulldorff’s purely spatial cluster analysis, based on the mean cases and mean population, indicated that the cases of mumps were not randomly distributed in space between 2005 and 2016. A total of 28 significant spatial clusters were detected using this method and 10 of them were listed in Table 3. The most likely cluster was mainly found in the southwestern part of Guangxi, including 11 counties (radius: 77.88 km) that scattered in the cities of Nanning, Chongzuo and Fangchenggang. The LLR and RR values were 350.67 and 1.79, respectively. The secondary cluster and the 2nd secondary cluster were located in the west and north of Guangxi, including 10 and 15 counties, respectively. The first three clusters included 40.74% of cases, although they accounted for 25.65% of the population at risk (Fig. 6).

**Table 3:** The clusters of mumps cases detected by using the purely spatial scan statistics

Cluster type	Radius (km^2^)	Cluster areas (n)	Observed	Expected	ODE	LLR	RR	*P* value
Most likely	77.88	11	2841	1713.33	1.66	350.67	1.79	< 0.001
Secondary	87.55	10	1832	1135.63	1.61	194.88	1.69	< 0.001
2nd Secondary	89.14	15	2405	1608.29	1.50	191.48	1.57	< 0.001
3rd Secondary	0.00	1	396	127.67	3.10	182.04	3.15	< 0.001
4th Secondary	0.00	1	693	317.24	2.18	169.89	2.23	< 0.001
5th Secondary	0.00	1	353	120.51	2.93	148.47	2.97	< 0.001
6th Secondary	11.44	3	412	194.44	2.12	93.19	2.15	< 0.001
7th Secondary	0.00	1	313	137.51	2.28	82.85	2.30	< 0.001
8th Secondary	51.14	2	503	270.94	1.86	80.72	1.88	< 0.001
9th Secondary	49.13	3	574	327.67	1.75	77.26	1.78	< 0.001

*ODE* observed/expected, *LLR* log likelihood ratio, *RR* relative risk

**Fig. 7:**
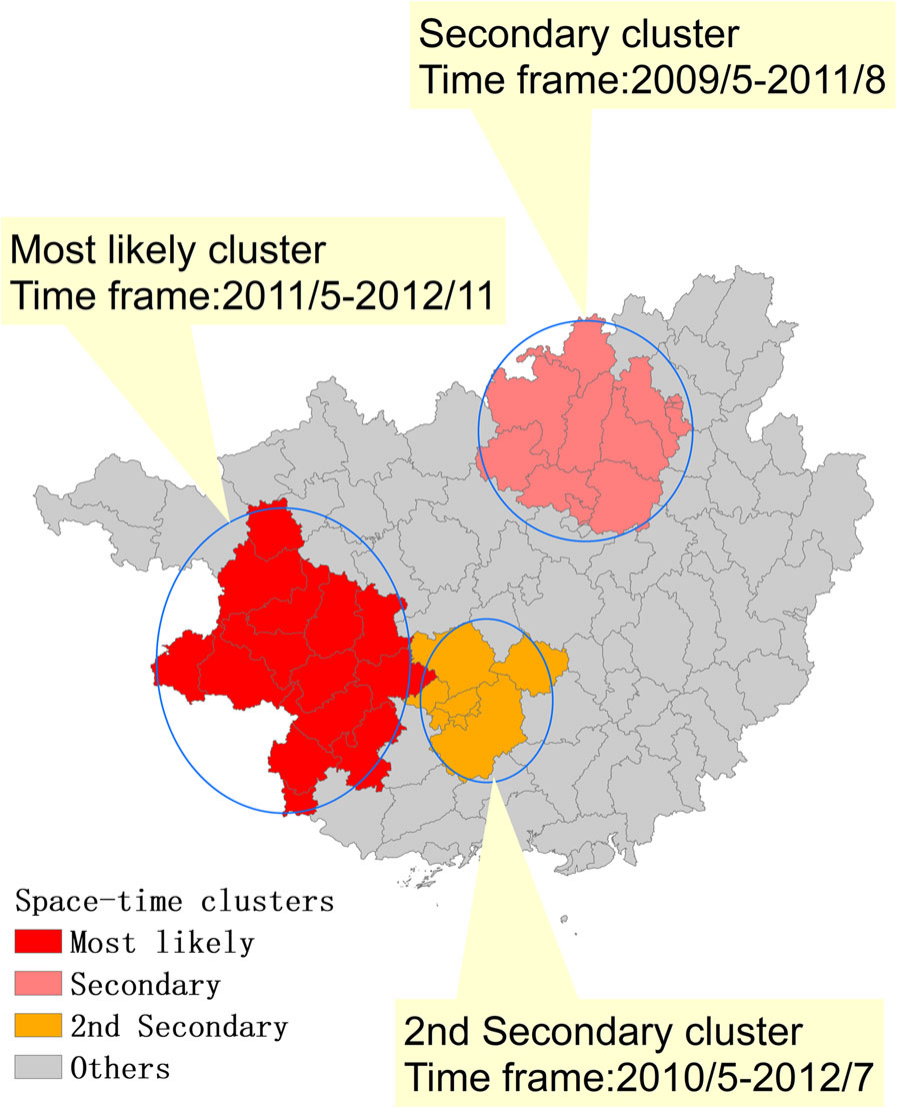
Spatiotemporal clusters of mumps at county level in Guangxi, China from 2005 to 2016

Kulldorff’s spatiotemporal scan statistics was applied to detect the spatiotemporal clusters in the entire study period. The top 10 clusters and their related attributes were listed in Table 4 and the first three clusters were illustrated in Fig. 7. The most likely cluster, located in the western part of Guangxi, included 14 counties, and the time frame was from May 2011 to November 2012. Similarly, the secondary cluster (*n* = 15) and the 2nd cluster (*n* = 8) were located in the northern and central regions of Guangxi, and the time frame were from May 2009 to August 2011 and from May 2010 to July 2012, respectively. Hence, the period between 2009 and 2012 was the high incidence of mumps. This result was consistent with the previous spatial autocorrelation results.

**Table 4:** The clusters of mumps cases detected by using the space-time scan statistics

Cluster type	Time frame	Radius (km^2^)	Cluster areas (n)	Observed	Expected	ODE	LLR	RR	P value
Most likely	2011/5–2012/11	140.70	14	8735	2463.95	3.55	4880.04	3.66	< 0.001
Secondary	2009/5–2011/8	89.14	15	11,050	3791.62	2.91	4691.20	3.02	< 0.001
2nd Secondary	2010/5–2012/7	66.71	8	10,862	3898.21	2.79	4286.84	2.88	< 0.001
3rd Secondary	2007/12–2009/4	21.08	3	2027	313.37	6.47	2077.72	6.52	< 0.001
4th Secondary	2010/5–2012/7	65.28	5	4178	1430.65	2.92	1748.52	2.96	< 0.001
5th Secondary	2008/5–2009/7	106.00	10	4406	1834.70	2.40	1304.78	2.43	< 0.001
6th Secondary	2011/4–2012/7	56.64	6	4359	1963.44	2.22	1094.87	2.25	< 0.001
7th Secondary	2009/3–2011/6	43.22	3	1679	562.82	2.98	721.96	3.00	< 0.001
8th Secondary	2011/12–2012/8	0.00	1	879	215.04	4.09	574.71	4.10	< 0.001
9th Secondary	2011/5–2011/7	40.27	3	759	321.71	2.36	214.66	2.36	< 0.001

*ODE* observed/expected, *LLR* log likelihood ratio, *RR* relative risk

## Discussion

This study clarified the basic epidemiological characteristics of mumps and illustrated its substantial changes occurring with respect to the spatial, temporal and spatiotemporal trends and clusters in Guangxi, China during 2005–2016, using GIS and scan statistics. Results showed that the annual incidence rate and the annual reported cases of mumps were declining as a whole, from 41.84(per 100,000) (19,338) in 2005 to 19.13(per 100,000) (9176) in 2016. This result may be due to the introduction of mumps vaccine in China in the 1990s. However, two outbreaks occurred in 2006 and 2011. Similar phenomena can be observed in other parts of China [17, 18]. Likely because of the periodic changes in the incidence of mumps. Before 2007, mumps vaccination was based on the principle of voluntary inoculation and self-paying inoculation. Given the limitations of medical resources and economic conditions in different regions, the coverage of vaccination is low, especially in the underdeveloped province of Guangxi. An effective antibody barrier did not form in the crowd and susceptible populations continued to accumulate, thereby leading to a cyclical outbreak of mumps [29]. MMR was included in the EPI by the Chinese government in 2008. Since then, children aged 18 and 24 months have been receiving a free dose of mumps vaccine. Supplementary vaccination and the second dose of mumps vaccination have also been implemented in some areas with high incidence. All of these interventions led to a rapid decline in the incidence in high-risk group of 5–9 years old after 2013, and the overall number of cases simultaneously decreased. This result suggested that the prevention and control for mumps had been effective to some extent.

The onset of mumps was mainly observed in students and childcare children in 5–9 years old. This finding is consistent with previous results [9, 22, 30]. Mumps is a respiratory disease, and adolescents and children are generally susceptible. Children aged 5–9 years old should attend kindergarten or elementary school in China. The low immunity of children, dense population, crowded environment and personal hygiene are important factors that cause its transmission. The increasing incidence of mumps among the elderly population is another matter of concern in this study. On the one hand, the vaccination coverage of mumps in this age group has remained low. Although the circulation incidence can provide immunity from natural infection to people, the elderly population has less opportunity to obtain such natural immunity because the occurrence of mumps epidemic gradually decreases as a result of the implementation of the national immunization planning policy and effective control of infectious diseases [31]. On the other hand, a constant attenuation of mumps antibodies is observed in human serum. Differences in antibody levels between populations may be induced by several factors, including uptake of mumps vaccine, different immunogenicity of mumps vaccines or variation in schedules, (including spacing between doses), and previous exposure to mumps infection. Even two doses of mumps vaccine will not achieve a 100% seroconversion; hence, a group of susceptible individuals remains in the population [32, 33]. In addition, previous studies found that mumps virus genotypes in different Chinese provinces vary, but the present mumps vaccine mainly targets the F type. The immunization effect between different F subtypes and other types of virus has yet to be evaluated [34]. All of these factors may increase the risk of infection in the elderly and children, suggesting that the enhanced immunization and supplementary vaccination should be implemented for these special populations.

In the current study, mumps cases were reported in all 12 months, but most occurred between April and July, with a small peak in October and January. This bimodal seasonal distribution was consistent with that of northern China [17, 18, 34] and prompts that there may be a relationship between the mumps incidence and the climate. A study in Guangzhou found that mean temperature, relative humidity, and atmospheric pressure can increase the risk of mumps, whereas wind speed has a protective effect [35]. Another study in Jining showed that temperature, humidity, and sunshine have a linear relationship with mumps incidence when exceeding a certain threshold [36]. Our previous research also indicated that meteorological factors, such as temperature and wind speed, can exert a significant impact on the mumps incidence, which can be modified by several socioeconomic factors (submitted). According to the seasonality of mumps incidence, local medical institutions should formulate and implement targeted intervention strategies before the advent of disease peak period.

The spatial analysis in this study demonstrated a significant spatiotemporal heterogeneity throughout Guangxi in 2005–2016. The results of the global autocorrelation analysis revealed a spatial positive correlation in the study period, whereas the annual analysis did not show the same trend. This phenomenon indicated that the high-incidence areas of mumps are unstable. The results of the LISA and purely spatial cluster analysis showed a remarkable variation in the spatial distribution of mumps in Guangxi, with most high-risk counties located in the cities of Liuzhou, Nanning, and Chongzuo. The counties where mumps constantly emerged were scattered in remote mountainous areas and in economically developed cities. With the increasing number of young people born in mountainous areas but leaving their hometowns to seek job opportunities in urban areas, many children are left at home supported by their grandparents. Given the remote distance, traffic congestion, economic backwardness, and serious brain drain, mountainous areas are characterized by poor access to health services, scarce health resources, and inadequate health care services for children. The timeliness and completeness of mumps vaccination are also not guaranteed [37]. This condition explains why some clusters are found in poor counties located in the western and northern parts of Guangxi, such as Jiangzhou, Xilin, and Luocheng. In addition, only a few children who migrate to the urban or suburban areas with their parents can timely receive free mumps vaccination [21, 38]. Meanwhile, the current immunization strategies do not allow local children to develop high antibody concentration. Hence, a disease outbreak may occur easily in labor-importing regions like Xingning, Wuming, Yufeng, and Liunan. Increasing the coverage of health services and implementing two or three doses of immunizations may be the focus of local health agencies.

The first three likely clusters identified using the spatiotemporal clustering analysis were located in the western, northern, and central parts of Guangxi, respectively, which were consistent with the hot spots of LISA. However, these high-risk areas were not the same as those of the purely spatial scan analysis possibly because of the difference in the analysis variables included in the two scanning methods. The temporal clusters identified using the spatiotemporal scan statistics were mainly recorded between 2009 and 2012. The trend of annual incidence and the seasonal pattern also indicated that the incidence of mumps was higher in this period than in other periods, suggesting a resurgence, which has yet to be examined for specific causes.

In addition, this study found that spatiotemporal analysis has unique advantages in detecting the high incidence of disease and determining the scope of prevention and control compared with the traditional methods of epidemiological analysis [39–41]. LISA has a better chance of detecting true cluster areas with low false-positive rates especially performing well on outlier detection [42]. Simultaneously, Kulldorff’s statistical analysis has a strong power to detect mumps spatial distribution and focus on the extent of mumps [43]. These two spatial analysis methods have been widely used in the field of public health. The use of a combination of these methods can enhances the comprehensive understanding of the spatial distribution patterns and characteristics of the disease [44–46].

The results of this study should be interpreted in consideration of the following limitations. First, data are obtained from a passive surveillance system in China, which means that some cases of mumps may have been underreported because of their sub-clinical symptoms, even though quality control with data collection has been an important component of the disease surveillance system in China for all reportable diseases [47]. Second, a circular scan window is used in the purely spatial and spatiotemporal scan statistics. Although a circular scan window requires less computational time and performs well at detecting for large clusters, it cannot effectively identify the narrow, long, and noncontiguous areas [48]. Finally, this study only analyzed the spatial distribution pattern and epidemiological characteristics of mumps between 2005 and 2016. The environmental factors, meteorological data, urbanization, and sociological factors that may drive the mumps epidemic have yet to be examined in detail. Thus, a flaw may exist in the interpretation of the causal relationship of the disease.

## Conclusions

This study, to our knowledge, is the first to described the epidemiological characteristics of mumps by using the spatiotemporal analysis at the county level in Guangxi, in southern China and identified the spatial and spatiotemporal high-risk clusters from 2005 to 2016. Though, the mumps incidence generally decreased to a low level, there still exist some high-risk areas of mumps, where mainly concentrated in the western, northern and central regions of Guangxi. Remote urban districts and densely populated urban areas may become mumps epidemic areas. Male children and children aged 5–9 years are susceptible to the mumps incidence in this study. Measures targeting these vulnerable groups and high-risk areas should be developed to prevent future mumps outbreak and reduce geographical inequities in the burden of mumps and allocation of health resources.

## Additional file

Additional file 1: **Figure S1.** The trend of mumps incidence in different age groups between 2005 and 2016 in Guangxi, China. (DOCX 202 kb)

## Abbreviations

CISDCP: China Information System for Disease Control and Prevention
EPI: the expanded program on immunization
GIS: Geographic information system
LISA: local indicators of spatial association
LLR: log likelihood ratio
MMR: measles–mumps–rubella vaccine
RR: relative risk
SEB: Spatial empirical Bayesian
SIAs: supplementary immunization activities
