# Supplementary material for: Spatial, temporal, and spatiotemporal analysis of mumps in Guangxi Province, China, 2005–2016
Source: BMC Infect Dis. 2018 Aug 2;18:360. doi: 10.1186/s12879-018-3240-4 (PMC6090846; doi:10.1186/s12879-018-3240-4)
Supplement: Supplementary file 1 — Figure S1. The trend of mumps incidence in different age groups between 2005 and 2016 in Guangxi, China. (DOCX 202 kb) [file 12879_2018_3240_MOESM1_ESM.docx]

**Figure S1.** The trend of mumps incidence in different age groups between 2005 and 2016 in Guangxi, China.
